# ZFIN updates to support zebrafish environmental exposure data

**DOI:** 10.1093/genetics/iyaf021

**Published:** 2025-02-04

**Authors:** Yvonne M Bradford, Ceri E Van Slyke, Jonathan B Muyskens, Wei-Chia Tseng, Douglas G Howe, David Fashena, Ryan Martin, Holly Paddock, Christian Pich, Sridhar Ramachandran, Leyla Ruzicka, Amy Singer, Ryan Taylor, Monte Westerfield

**Affiliations:** The Institute of Neuroscience, University of Oregon, 5291 University of Oregon, Eugene, OR 97403-5291, USA; The Institute of Neuroscience, University of Oregon, 5291 University of Oregon, Eugene, OR 97403-5291, USA; The Institute of Neuroscience, University of Oregon, 5291 University of Oregon, Eugene, OR 97403-5291, USA; The Institute of Neuroscience, University of Oregon, 5291 University of Oregon, Eugene, OR 97403-5291, USA; The Institute of Neuroscience, University of Oregon, 5291 University of Oregon, Eugene, OR 97403-5291, USA; The Institute of Neuroscience, University of Oregon, 5291 University of Oregon, Eugene, OR 97403-5291, USA; The Institute of Neuroscience, University of Oregon, 5291 University of Oregon, Eugene, OR 97403-5291, USA; The Institute of Neuroscience, University of Oregon, 5291 University of Oregon, Eugene, OR 97403-5291, USA; The Institute of Neuroscience, University of Oregon, 5291 University of Oregon, Eugene, OR 97403-5291, USA; The Institute of Neuroscience, University of Oregon, 5291 University of Oregon, Eugene, OR 97403-5291, USA; The Institute of Neuroscience, University of Oregon, 5291 University of Oregon, Eugene, OR 97403-5291, USA; The Institute of Neuroscience, University of Oregon, 5291 University of Oregon, Eugene, OR 97403-5291, USA; The Institute of Neuroscience, University of Oregon, 5291 University of Oregon, Eugene, OR 97403-5291, USA; The Institute of Neuroscience, University of Oregon, 5291 University of Oregon, Eugene, OR 97403-5291, USA

**Keywords:** zebrafish, *danio rerio*, environmental exposure, phenotype, human disease model, expression, ZFIN, model organism database

## Abstract

The Zebrafish Information Network (ZFIN, zfin.org) is the database resource for genetic, genomic, and phenotypic data from research using zebrafish, *Danio rerio*. ZFIN curates information about genetic perturbations, gene expression, phenotype, gene function, and human disease models from zebrafish research publications and makes these data available to researchers worldwide. Over the past 20 years, zebrafish have increasingly been used to investigate the effects of environmental exposures, becoming an ideal model to study toxicity, phenotypic outcomes, and gene-chemical interactions. Despite this, database resources supporting zebrafish toxicology and environmental exposure research are limited. To fill this gap, ZFIN has expanded functionality to incorporate and convey toxicology data better. ZFIN annotations for gene expression, phenotype, and human disease models include information about genotypes and experimental conditions used. One type of experimental condition the database captures is the application of chemicals to zebrafish. ZFIN annotates chemicals using the Chemical Entities of Biological Interest Ontology (ChEBI) along with the Zebrafish Experimental Conditions Ontology (ZECO) to denote route of exposure and other experimental conditions. These features allow researchers to search phenotypes and human disease models linked to chemicals more efficiently. Here, we discuss how experimental conditions are displayed on ZFIN web pages, the data displayed on chemical term pages, and how to search and download data associated with chemical exposure experiments.

## Introduction

Chemical exposure affects all forms of life and can have effects on developmental processes, health, and disease. Toxicology and environmental exposure research helps scientists and clinicians understand how exposure to xenobiotics affects biological processes and contributes to disease states. Zebrafish have long been used as a model organism to study vertebrate biology and is an excellent system to model human disease etiology due to the conservation between human and zebrafish genomes, in addition to similarities in physiology (Howe *et al*. 2013; Postlethwait *et al*. 2000). Based on these parallels to humans, zebrafish are positioned to provide insights into the interplay between the genome and chemical exposures and, thus, to help develop a better understanding of gene–environment interactions and how these interactions lead to phenotypic outcomes. Over the past several decades, zebrafish have become widely used in toxicology and chemical exposure studies to evaluate the effect of environmental exposures and ecotoxicity (Bambino and Chu 2017), chemical toxicity screening (Kaufman *et al*. 2009; Rennekamp and Peterson 2015), drug discovery and development (Zon and Peterson 2005; Gibert *et al*. 2013; Cassar *et al*. 2020), developmental neurotoxicity (Bailey *et al*. 2013; Nishimura *et al*. 2015), and developmental toxicity (Alzualde *et al*. 2018). Despite the large-scale use of zebrafish in such studies, there is limited database support for zebrafish toxicology and chemical exposure data.

The Zebrafish Information Network (ZFIN) is bridging this gap by providing annotation and curation of zebrafish xenobiotic data. ZFIN serves as the knowledgebase resource for zebrafish research by annotating, curating and making data available from zebrafish research publications (Bradford *et al*. 2022). ZFIN annotates information about gene mutants, gene expression, orthology, phenotype, and human disease models (Sprague *et al*. 2008; Ruzicka *et al*. 2015; Howe *et al*. 2017). These annotations include experimental condition details such as chemicals applied and route of exposure. Here, we discuss how we annotate chemicals in experimental conditions, the ZFIN webpages to view and search these data, download files for bulk data use, as well as links out to the Comparative Toxicogenomic Database (CTD, https://ctdbase.org/), for users to gather more gene-chemical information.

### Annotation of chemical experiments

**Fig. 1.**
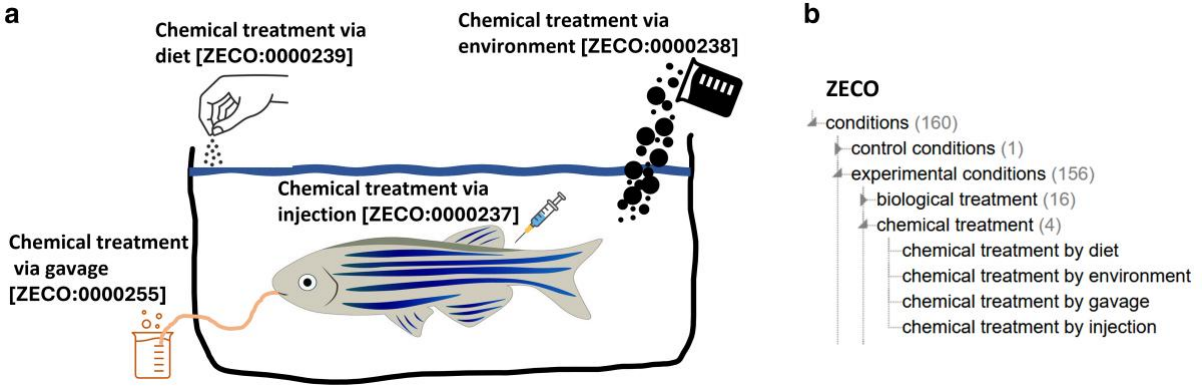
Zebrafish experimental conditions ontology (ZECO) describes a) Four routes of exposure of toxins/chemicals to zebrafish. b) Hierarchical structure of ZECO chemical exposure terms. Note that the parent term “chemical treatment” can be used when specific routes of exposure are unspecified by authors.

**Fig. 2.**
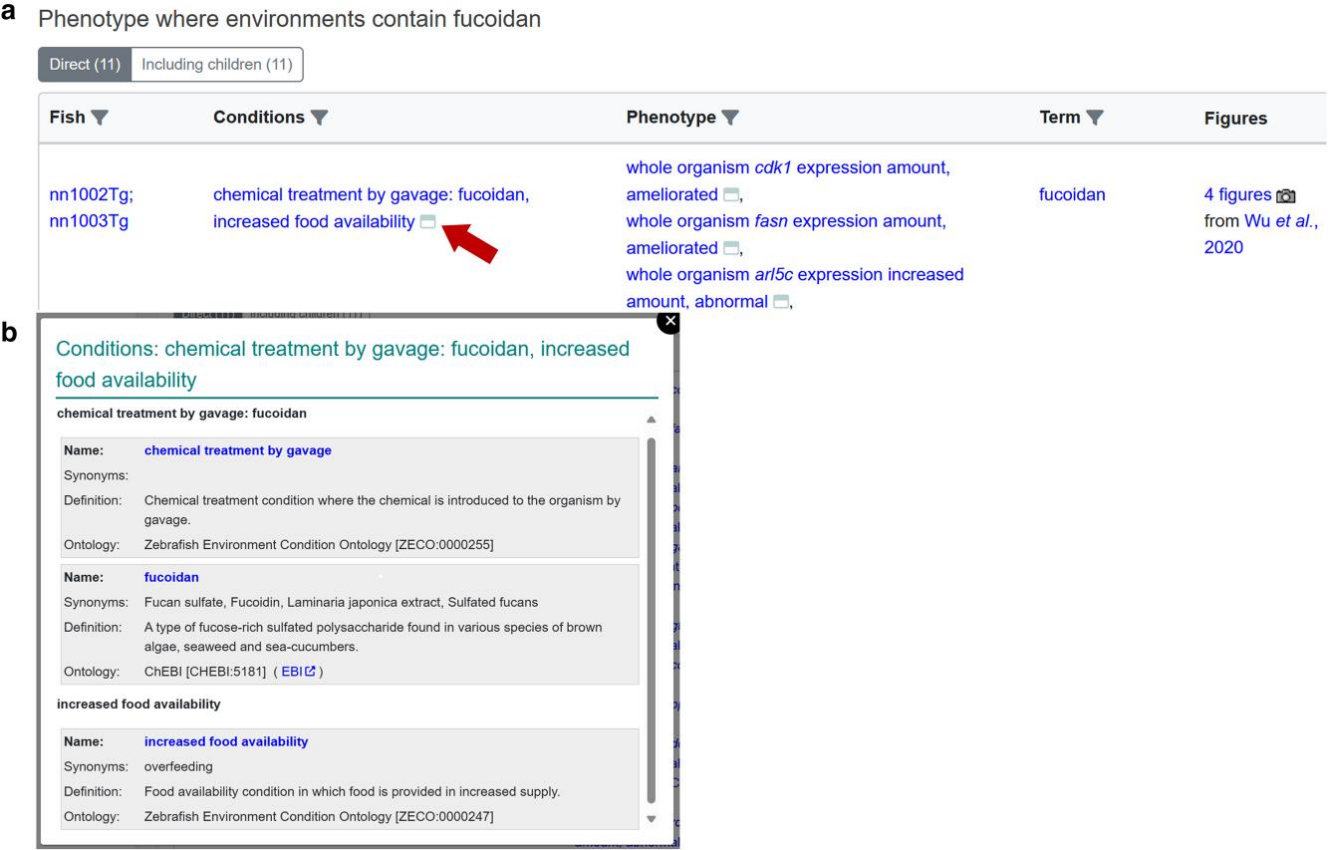
Multiple experimental conditions annotation. a) Example of phenotype annotation using multiple environmental conditions. Arrow denotes icon to access pop-up. b) The pop-up lists all terms used in the condition annotation with their definitions. https://zfin.org/ZDB-FIG-210103-23#phenotype-data.

Chemicals are used in zebrafish experiments in many ways. For example, they have been used to model the effects of diseases such as Rubinstein–Taybi syndrome by treating fish with C646 (Babu *et al*. 2018), to study the effects of long-term chemical exposure on development (Liu *et al*. 2016), or to explore the interactions of chemicals with genetic variants (Vujica *et al*. 2024). To capture salient features of chemical exposure experiments, such as the method of exposure and the chemical applied, ZFIN uses the Zebrafish Experimental Conditions Ontology (ZECO) (Bradford *et al*. 2016) and the Chemical Entities of Biological Interest (ChEBI) (https://www.ebi.ac.uk/chebi/; Hastings *et al*. 2016), respectively. The most common method to treat a zebrafish with a chemical is to add it to the water in which the fish is placed. This concept is represented in ZECO by the term “chemical treatment by environment” [ZECO:0000238] (Fig. 1a). Examples of other chemical application methods include (1) adding the chemical to the food, represented by the term “chemical treatment by diet” [ZECO:0000239]; (2) injecting the chemical, represented by “chemical treatment by injection” [ZECO:0000237]; and (3) by gavage, represented by “chemical treatment by gavage” [ZECO:0000255] (Fig. 1a). Previously, experimental conditions were captured as free text. When ZFIN converted to using ontologies for experimental conditions, we identified experimental conditions with chemicals computationally using Chemical Abstract Service (CAS) identifiers noted in the text and applied the ZECO parent term “chemical treatment” with the appropriate ChEBI term to avoid manual back curation of route of exposure. On the rare occasion, curators are unable to determine the treatment route from the publication, they follow standard ontological practice using the parent term “chemical treatment” [ZECO:0000111] (Fig. 1b). The chemical treatment ZECO terms are always associated with a ChEBI chemical (Fig. 2). During the course of curation, ZFIN curators actively contribute chemicals to the ChEBI ontology and the ChEBI ontology files used for curation in ZFIN are updated with ChEBI monthly releases, ensuring newly added terms or changes to ChEBI are available for use in ZFIN curation and web interfaces.

Using ontologies to annotate route of exposure and chemical applied provides ZFIN great flexibility in recording exactly how chemicals are used in experiments as well as annotating experiments involving multiple chemicals and conditions. This is important because zebrafish are often used to explore synergistic or muting effects of multiple chemicals in a controlled pairwise fashion (Carroll *et al*. 2014) or in environmental samples (McElroy *et al*. 2012). In an annotation of such an experiment, the combined set of treatments is described in the “conditions” column in the gene expression, phenotype, and human disease sections of ZFIN. Figure 2a is an example where the authors treated the fish with fucoidan via gavage and modulated food availability. Clicking the icon at the end of the conditions reveals the terms that describe the experiment together with their ontology IDs (Fig. 2b).

### CTD mapping

**Fig. 3.**
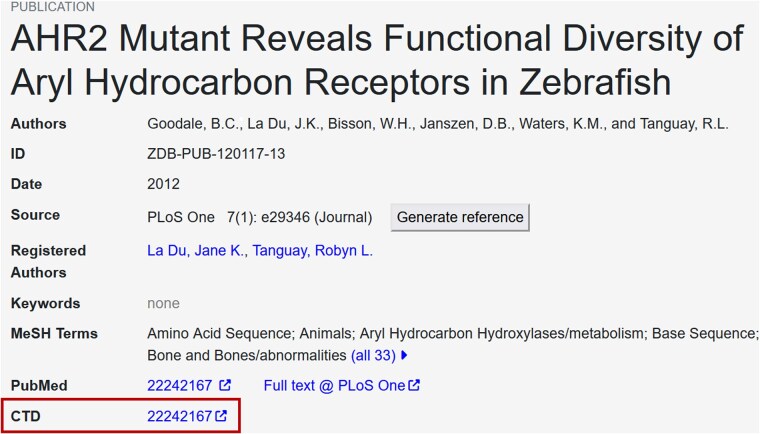
Publication detail page. ZFIN publication page summary section with link to CTD publication page (red box). Clicking the link redirects to the corresponding page at CTD. https://zfin.org/ZDB-PUB-120117-13.

To provide ZFIN users access to a larger set of curated toxicology data, including other organisms, ZFIN links to CTD from publication and chemical term pages. CTD is a curated database of harmonized cross-species chemical exposure data that provides insights into the effects of environmental exposure on human health (Davis *et al*. 2009). CTD manually curates chemical exposure data for 622 organisms, including zebrafish, and includes information about chemicals, genes, phenotypes, anatomy, disease, and taxa (Davis *et al*. 2023). ZFIN utilized CTD query interfaces and download files (https://ctdbase.org/downloads/) to obtain a list of publications that contain zebrafish specific data. This list was then cross referenced to the 8,500 ZFIN toxicology publications to generate a mapping file that is used to provide links from ZFIN publication pages to CTD publication pages (Fig. 3). ZFIN updates links monthly. As of October 2024, there were 1,241 publications in ZFIN that have a link to a corresponding publication page at CTD.

**Fig. 4.**
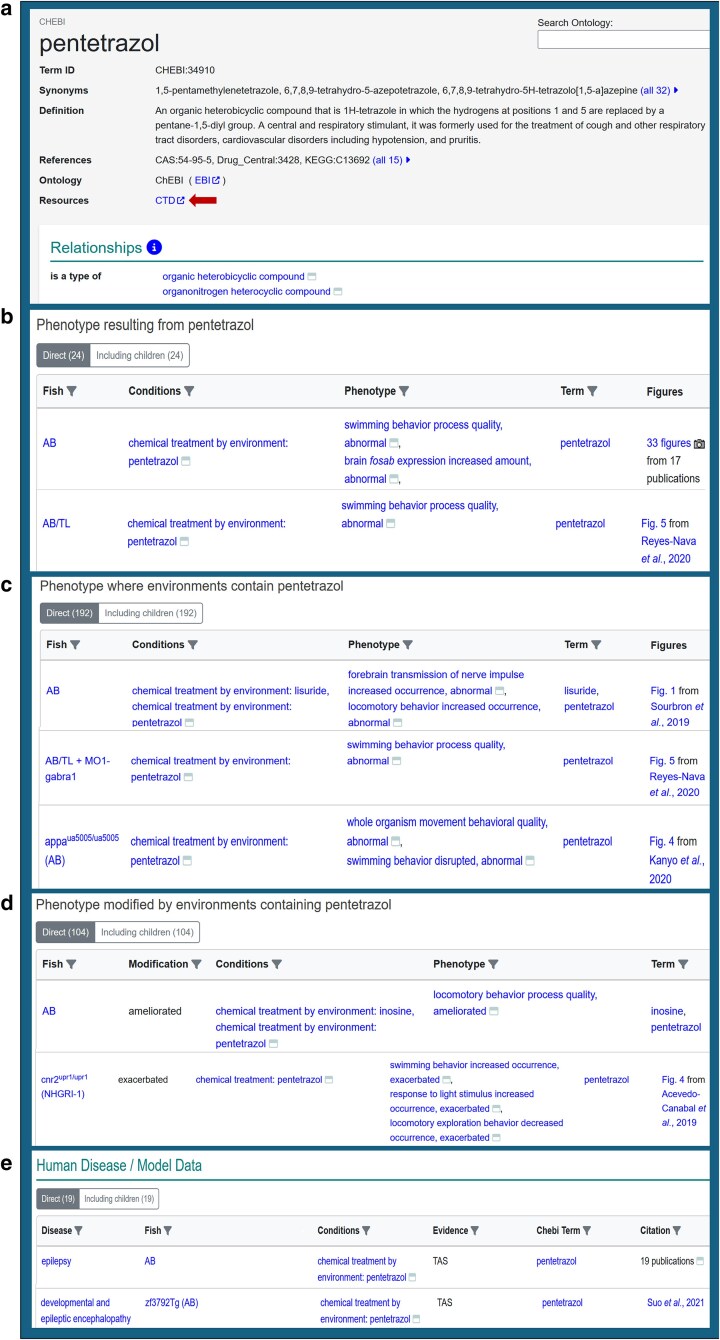
Pentetrazol chemical term page. a) Summary section of chemical term page. This provides term information such as synonyms and cross-references to other resources. Arrow denotes links to corresponding CTD page. b) First table of Phenotype section “Phenotype resulting from pentetrazol”. Table aggregates phenotypes inferred to be caused by pentetrazol. c) Second table of Phenotype section “Phenotypes where environments contain pentetrazol”. Table aggregates all phenotypes that have an experimental condition which is annotated with pentetrazol. d) Third table of Phenotype section “Phenotype modified by environments containing pentetrazol”. Table aggregates all phenotypes that have an experimental condition that is annotated with pentetrazol and are either ameliorated or exacerbated. e) Human disease section of chemical term page. This table lists human disease model annotations that have conditions containing pentetrazol. Evidence column lists either traceable author statement (TAS) or inferred by curator (IC) codes that indicate that there is evidence from a publication that directly supports the disease model annotation or the annotation was made on the basis of curatorial judgement, respectively. https://zfin.org/CHEBI:34910.

In addition to linking to CTD from publication pages, ZFIN provides links to CTD from chemical term pages (Fig. 4a). ZFIN uses ChEBI terms to annotate chemical experiments. In contrast, CTD uses a subset of the Chemicals and Drugs branch of the National Library of Medicine's Medical Subject Headings (MeSH) (Coletti and Bleich 2001) vocabulary for the chemical terms used in their annotations (Davis *et al*. 2011). To link effectively to CTD chemical pages from ZFIN chemical pages, we generated a MeSH-ChEBI mapping file using the CTD chemical vocabulary file (https://ctdbase.org/downloads/#allchems) and the ChEBI ontology file. The mapping logic uses the CAS identifiers specified for the chemicals in each file and the Simple Standard for Sharing Ontology Mappings (SSSOM) format (Matentzoglu *et al*. 2022). The MeSH-ChEBI mapping file is updated monthly and is available from the publicly available ZFIN download files (https://zfin.org/downloads/mesh-chebi-mapping.tsv), as well as from Zenodo (Pich *et al*. 2024).

ZFIN will continue to work with CTD to align gene–chemical interactions to provide these data on ZFIN gene, chemical, and publication pages, as well as ZFIN providing chemical interaction data to CTD and align phenotype data between these resources.

### Chemical term pages

To support better visualization of data related to zebrafish chemical exposure research, chemical term pages have been created in ZFIN to display general chemical term information and annotations that have chemicals in experimental conditions. The chemical term page starts with a summary section that provides the ChEBI term name, synonyms, definitions, cross references, and links to ChEBI and CTD (Fig. 4a). The phenotype section has 3 tables that report the phenotypes that result from the application of the chemical alone or in combination with other chemicals or in mutant strains (Table 1).

**Table 1.** Chemical term page phenotype table descriptions.

Table name	Single ChEBI term	ChEBI term + multiple conditions	Only WT genotype	All fish: mutant, transgenic and WT genotypes	Description
Phenotype resulting from chemical X	✓		✓		Phenotype resulting from wild-type zebrafish treated with only chemical X
Phenotype where environments contain chemical X		✓		✓	Phenotype resulting from wild-type, mutant/transgenic, morpholino or CRISPR treated fish treated with chemical X and/or other conditions
Phenotype modified by environments containing chemical X	✓	✓		✓	Phenotype ameliorated or exacerbated in wild-type, mutant/transgenic, morpholino or CRISPR treated fish treated with chemical X and/or other conditions

The first table represents the simplest case, the effect of a single chemical on a wild-type fish. This table labeled “Phenotype resulting from chemical X’, reports phenotype annotations where the chemical is inferred to be causative (Fig. 4b). The data for this table are aggregated using similar algorithms that were developed to determine whether a single gene is causative of experimental outcomes (Bradford *et al*. 2023). These algorithms were modified to report whether a chemical can be inferred to be causative for phenotypic outcomes by determining whether the annotation referenced a wild-type genotype, and the only experimental condition is a single chemical. One of the strengths of ZFIN experiment models and rigorous recording of genetic backgrounds is that these attributes of the annotation can be used to determine computationally whether a chemical can be inferred to be causative of a phenotype.

Many chemical experiments use multiple chemicals or genetic backgrounds, or the phenotype is modified in some other way. Two additional tables in the phenotype section display these data. The second table labeled “Phenotype where environments contain chemical X” displays the annotations that include a wild-type or mutant fish and the experimental conditions include treatment with the chemical and any other treatments (Fig. 4c). The table provides an overview of the various ways the chemical has been used in experimental conditions and the associated phenotypic outcomes. The final table in the phenotype section is the “Phenotype modified by environments containing Chemical X” (Fig. 4d). This table displays phenotypes that are ameliorated or exacerbated by the chemical and is useful for understanding the potential for the chemical as a treatment for phenotypic outcomes or if it acts synergistically to enhance phenotypes.

The last section of the chemical term page is the human disease model section that reports human disease model annotations that have the chemical in the experimental conditions (Fig. 4e). These annotations are instances when a chemical is applied singularly to model a human disease, such as zebrafish that are treated with alloxan to model diabetes (Castañeda *et al*. 2017) (Hong *et al*. 2017; Nam *et al*. 2017) or are used in conjunction with mutant fish to model a human disease, for example, *slc39a14*^u801/u801^ mutants treated with manganese(II) chloride to model Parkinson's disease (Tuschl *et al*. 2016). Chemicals can also be applied to disease models to test if the chemical ameliorates or exacerbates a disease phenotype, providing potential avenues for treatment (Kawahara *et al*. 2011). Disease models with ameliorated phenotypes are displayed in the Phenotype modified by environments containing Chemical X’ table as described previously.

Chemical term pages serve as a summary of all information curated for experiments using a chemical. The intent is to give an overview of the current research and facilitate conclusions about the chemical's effect on zebrafish. With the inclusion of disease models and the table of phenotypic modifications, we hope to support studies of chemicals to treat disease. Future collaborations with CTD will enhance the content of the chemical pages while increasing their usefulness.

### Search support for chemical data

**Fig. 5.**
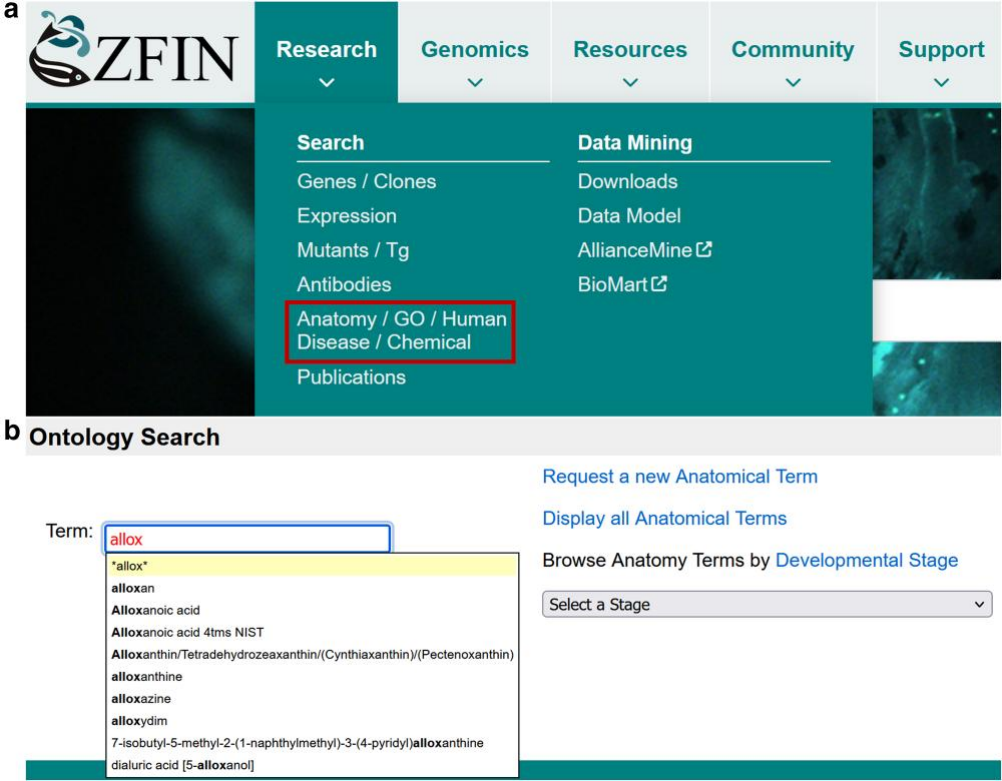
Ontology search. a) Ontology Search (red box) is available from the Research tab on the home page. Clicking the link redirects to the ontology search interface. b) Ontology search interface has a type ahead autocomplete text entry box to support searching ChEBI terms. https://zfin.org/action/ontology/search.

Users can search for ChEBI terms using the ontology search interface or faceted search. ZFIN has added the ability to search for ChEBI terms in the ontology term search, Anatomy/GO/Human Disease/Chemical, found in the Research tab (Fig. 5a). This search interface provides a simple entry point to search the Zebrafish Anatomy Ontology (ZFA; Van Slyke *et al*. 2014), Gene Ontology (GO; Ashburner *et al*. 2000; The Gene Ontology Consortium 2019), Disease Ontology (DO; Schriml *et al*. 2019), and the ChEBI Ontology. The term search box is type-ahead, auto-complete, and provides a drop-down list of matching terms (Fig. 5b). Picking from the list redirects the user to the appropriate term page.

Chemical names can also be searched in the faceted search, available from the center of the home page or the top right of all ZFIN pages. Faceted search enables the user to refine a search using a desired set of parameters. The faceted search features an autocomplete function that provides a list of options to choose from based on entered text. Selecting from the list of options takes the user to the selected page. If a “contains” search is desired, after text entry, one can click either the “Search” button on the home page or the magnifying glass icon at the top right of data pages to be taken to the faceted search results page.

**Fig. 6.**
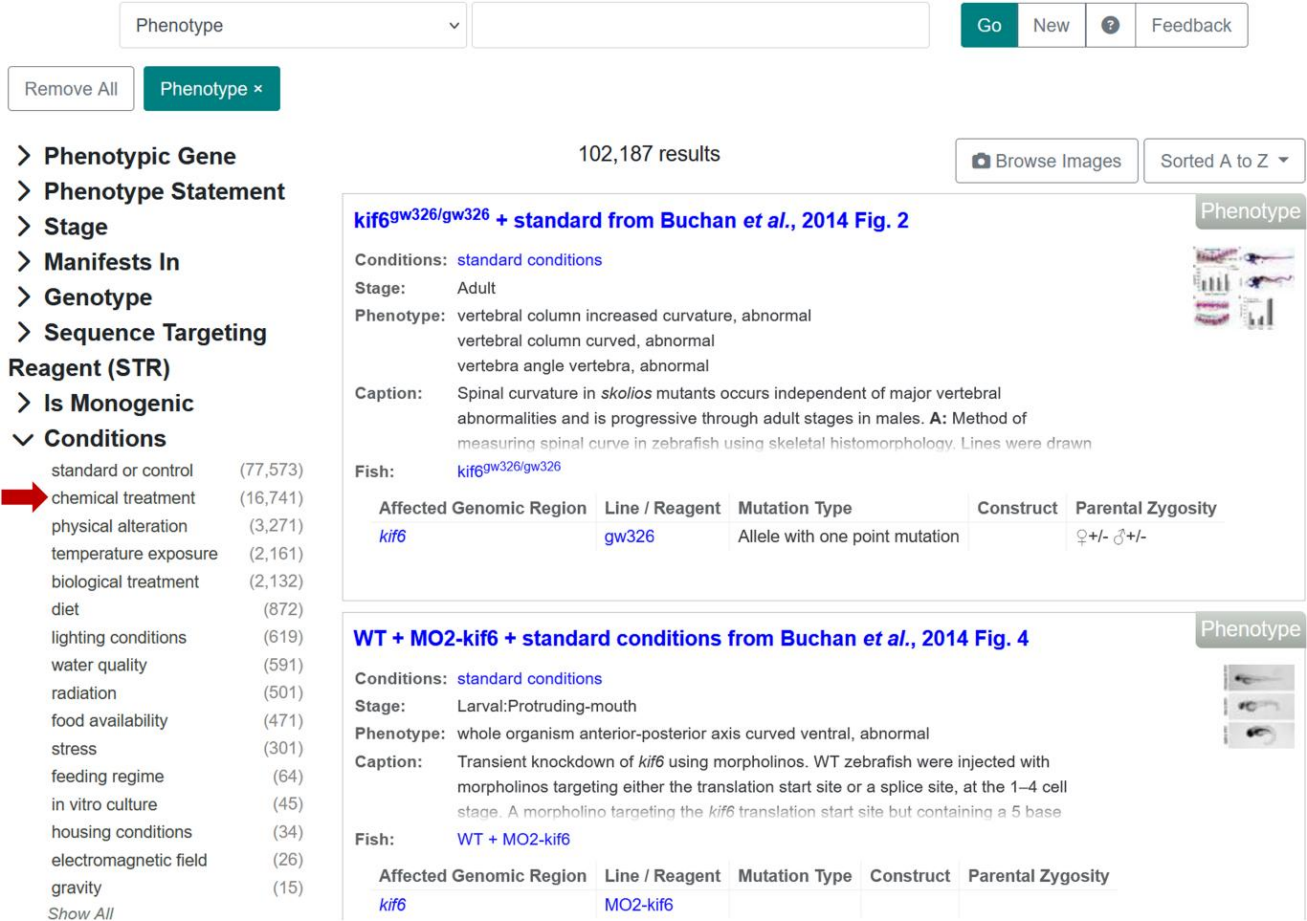
Faceted search results page. Phenotype search using faceted search. Facets on left allow users to select parameters to modify search results. Arrow denotes facet users can pick to refine phenotype search results to those annotations with a chemical condition. https://zfin.org/search?q=.

**Fig. 7.**
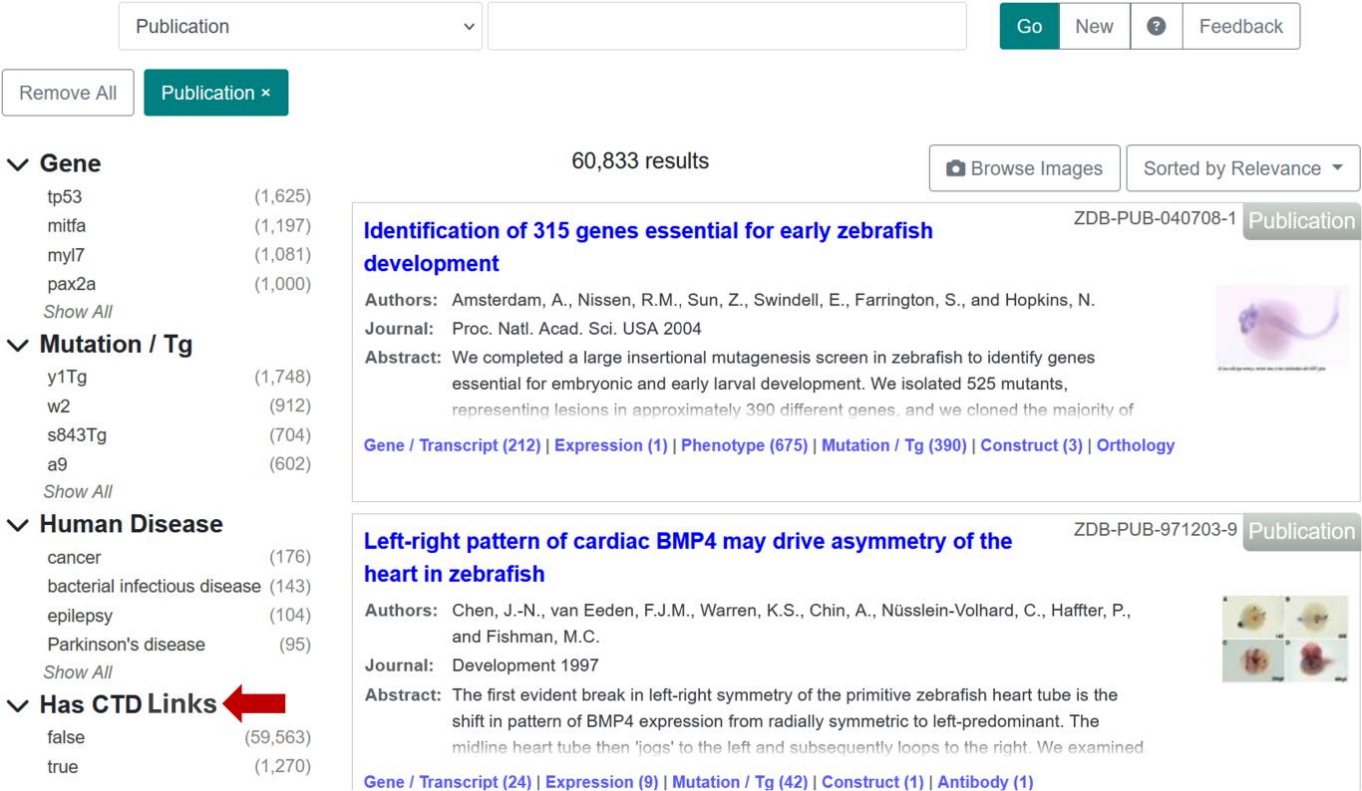
CTD facet in the publication category. The Publication category of faceted search has a CTD facet (arrow) to limit results to publications in ZFIN corpus that are linked to CTD. https://zfin.org/search?q=&fq=category%3A%22Publication%22&category=Publication&fq=has_ctd%3A%22true%22.

Information related to the effect of chemicals on gene expression, phenotype, or disease can be found also using ZFIN's faceted search interface. To query experiments involving chemical treatment, select the term “Chemical Treatment” found in the “Conditions” facet available in the Expression, Phenotype, or Human Disease Category (Fig. 6). Users can further refine search results by selecting from an array of parameters in the facets on the left, such as gene, anatomical feature, stage, and others. In addition, a new facet “Has CTD links” in the Publication Category, which uses mappings between the ZFIN publication corpus and CTD corpus described earlier, enables users to find publications that have been curated by CTD (Fig. 7).

ZFIN provides multiple search types to support many types of inquiries. The ontology search is useful for those who want to search for a specific chemical whereas the faceted search offers a means for users to refine results or to browse chemical data across multiple data types. As ZFIN incorporates additional chemical experiment data or additional CTD data, those data will be integrated into the appropriate section of faceted search.

### Toxicology download files

ZFIN provides download files to allow users to access bulk data (https://zfin.org/downloads). To support users who are interested in the effects of chemicals on zebrafish, we provide a category in the downloads files labeled “Toxicology” with 7 files that are updated daily (Table 2). For simplicity, the section is named “Toxicology”, although these files contain data on the effects of any chemical on zebrafish, including compounds that have therapeutic potential.

**Table 2.** Description of toxicology download files.

Download file name	All fish: mutant, transgenic and WT genotypes	Only WT genotype	ChEBI single term	ChEBI multiple terms	Non-chemical terms included in environment	Description
Ameliorated phenotypes with chemicals	✓			✓		File provides phenotypes that are ameliorated by the addition of a chemical.
Disease model that include chemical	✓			✓	✓*^a^*	File provides annotations of zebrafish disease models that are either created by the addition of the chemical or the chemical works in conjunction with a mutation to create the disease model.
Expression phenotypes with single chemical in environment in WT fish		✓	✓			File provides annotations that report gene expression changes in wild-type fish that result from the application of a single chemical.
Gene expression phenotypes with chemicals	✓		✓	✓	✓*^a^*	File provides annotations that report changes in gene expression in any genotype that have chemicals in the environment.
Phenotypes modified by chemical—ameliorated or exacerbated	✓			✓		File provides phenotypes that are ameliorated or exacerbated by the addition of a chemical.
phenotypes with single chemical in environment in WT fish		✓	✓			File provides phenotypes that result from the application of a single chemical in wild-type fish.
Phenotypes with chemical experiments	✓		✓	✓	✓	File provides all phenotype annotations that have a chemical in the environment.

^a^File provides all ZECO terms in annotation, but any other ontology terms used with those terms can be found in the “Expression Environment Description” download file.

To provide data from experiments involving exposure to a single chemical in wild-type fish, we created 2 downloadable data files: “Expression Phenotypes with Single Chemical in environment in WT fish”, and “Phenotypes with single chemical in environment in WT fish” (Table 2). The first file contains data related to the effect of a single chemical on the expression of a gene or protein in a specific anatomical region at a particular stage. The second file contains non-expression phenotype data related to the effect of single chemicals on a particular anatomical region at a specific stage. These files aggregate data collected in non-mutant strains and that have no other experimental conditions, thus it can be inferred that the resulting changes in gene expression and phenotype are caused by the chemical. These data can be used to help understand the pathways and processes on which the chemical acts.

ZFIN also provides downloadable files containing data on the interactions of chemicals with mutant backgrounds. The file “Gene Expression Phenotypes with Chemicals” contains data related to the effects of chemical exposure on the expression of genes and proteins in both wild-type and mutant backgrounds. Similarly, the file “Phenotypes with Chemical experiments” contains data related to the phenotypes resulting from chemical exposure in both wild-type and mutant backgrounds. These files provide all the gene expression and phenotype annotations that have chemicals in the experimental conditions. ZFIN also captures data from chemical exposure experiments that demonstrate either an amelioration or exacerbation of a phenotype resulting from a mutation or a second (or more) chemical exposure. Such chemical-gene or chemical-chemical exposure data are contained in the files, “Ameliorated Phenotypes with Chemicals” and “Phenotypes Modified by Chemical—Ameliorated or Exacerbated”. The “Ameliorated Phenotypes with Chemicals” file lists only phenotypes that have been ameliorated, or rescued, and can provide information about the chemical's potential for drug discovery or therapeutic treatment. The “Phenotypes Modified by Chemical-Ameliorated or Exacerbated” lists phenotypes that have been ameliorated or exacerbated and can be used to find synergistic or antagonistic chemical interactions to probe the effects they have on biological pathways.

Support for zebrafish disease models has expanded to include a downloadable file “Disease Models that include Chemical.” This file contains annotations of zebrafish human disease models where a chemical is used to model disease. These data provide information on the current state of the use of chemicals to create human disease models in zebrafish and offer examples of experimental models used for drug discovery or therapeutic treatment. In addition, these data can be used to find synergistic or antagonistic chemical interactions to improve the efficacy of drugs and explore the effects they have on biological pathways.

The chemical download files make the annotations for expression, disease, and phenotype affected by chemicals readily available for use both by researchers who would like to analyze the effects of a chemical and for organizations that aggregate chemical data. The data are freely available following the FAIR principles, which encourage data re-analyses, meta-analysis, as well as wide dissemination of the information to interested parties (Wilkinson *et al*. 2016). In addition, ZFIN data are available at the Alliance of Genome Resources (Alliance of Genome Resources Consortium 2020).

## Conclusion

Zebrafish have become one of the go-to model organisms to investigate chemical–gene interactions, perform chemical screens, and understand the outcomes of environmental exposures. As the knowledgebase resource for zebrafish genetic, genomic, and phenotypic research, ZFIN has expanded its curation and data representation to support phenotype, disease model, and gene expression annotations that report data for zebrafish that are treated with chemicals. These data can be visualized on newly created pages for ChEBI terms that display the annotations made to the term or children of the term as well as disease models and can be searched using ZFIN search interfaces. ZFIN will continue to collaborate with CTD to increase interoperability of data between these 2 resources. The increased support for environmental exposure data and the ability to access these data in combination with genetic and phenotypic data bring increased functionality to ZFIN users, enabling the synthesis of disparate data types to understand gene–chemical interactions, explore the effects of specific chemicals on gene pathways, and develop zebrafish models to study human diseases and potential therapeutics.

## Data Availability

All relevant data are available at ZFIN, zfin.org.
